# Social defeat stress causes selective attenuation of neuronal activity in the ventromedial prefrontal cortex

**DOI:** 10.1038/s41598-019-45833-5

**Published:** 2019-07-01

**Authors:** Reimi Abe, Sakura Okada, Ryota Nakayama, Yuji Ikegaya, Takuya Sasaki

**Affiliations:** 1 Laboratory of Chemical Pharmacology, Graduate School of Pharmaceutical Sciences, The University of Tokyo, 7-3-1 Hongo Bunkyo-ku, Tokyo, 113-0033 Japan; 2 Precursory Research for Embryonic Science and Technology, Japan Science and Technology Agency, 4-1-8 Honcho, Kawaguchi, Saitama 332-0012 Japan; 3 Center for Information and Neural Networks, Suita City, Osaka 565-0871 Japan

**Keywords:** Social behaviour, Stress and resilience

## Abstract

The ventromedial prefrontal cortex (vmPFC) plays key roles in higher cognitive abilities, including mental representations and the regulation of emotion. Previous studies have reported that vmPFC activity is altered in depressed human patients, highlighting this subregion as a major site of dysfunction in neuropsychiatric diseases. To examine how neuronal activity at spike levels in the vmPFC is altered by social defeat stress, we performed electrophysiological multiunit recordings along the dorsoventral axis of the mPFC of freely moving mice. Chronic social defeat stress-susceptible mice showing an impairment in social interaction exhibited significant reductions in the overall spike frequencies of neurons in the vmPFC, but not in the dorsal mPFC. Analysis of local field potentials revealed that the vmPFC generated spatially constrained 20–40 Hz events lasting hundreds of milliseconds, with an average event frequency of 0.05 Hz; during these events, a subset of neurons were transiently inhibited. The frequency of 20–40 Hz events in the vmPFC was reduced in defeated stress-susceptible animals, and this decrease was reversed by systemic ketamine administration. The novel neurophysiological correlates of stress-induced changes in the vmPFC advance the understanding of the neural basis of stress-induced dysregulation of social behavior.

## Introduction

The ventromedial prefrontal cortex (vmPFC) of rodents, the functional homolog of Brodmann area 25 of primates^1,2^, plays key roles in the regulation of affective and visceral functions^3–5^. The vmPFC preferentially projects to limbic and associative areas such as the basomedial amygdala and the ventral striatum, the hypothalamus and the brainstem^6–8^. It is distinct from the dorsal portion of the medial prefrontal cortex (dmPFC), which mainly innervates motor areas, sensory areas, and the basolateral amygdala and supports working memory, fear-related freezing, and the control of actions^4,8–10^. In animals subjected to chronic stress, vmPFC neurons exhibit pronounced dendritic atrophy^11–14^ and dysfunction of synaptic transmission^15^. The results of these animal studies are consistent with clinical evidence showing that human patients with mood disorders display gray matter loss, changes in molecular expression and blood-oxygen-level-dependent signaling specifically in the vmPFC^16–19^. Consistently, depression-like states and anxiety-related behavior can be reduced by vmPFC stimulation in both animals and humans^20–24^. Furthermore, recent electrophysiological studies have demonstrated that neuronal activity patterns in the PFC region and related brain areas in a pre-stressed state are associated with subsequent stress susceptibility^25–27^. Taken all, these studies highlight the vmPFC as a key brain region involved in the regulation of emotional states and neuropsychiatric pathology.

Although evidence for a relationship between the vmPFC and mental stress has been presented at both the molecular level and the cognitive behavioral level, few studies have directly assessed the neurophysiological characteristics of the vmPFC at the single-neuron level in living animals. Early pioneering works using single-unit recording from the infralimbic (IL) region have reported footshock stress-induced and restraint stress-induced changes in firing rates of neurons in the IL region during fear extinction^28,29^. However, no studies have assessed how social stress-induced pathophysiological changes in neuronal activity occurred specifically in the vmPFC by identifying detailed recording locations in the subregions of the PFC.

To address these questions, we simultaneously recorded extracellular signals, including local field potential (LFP) signals and multiunit spikes, from the dorsal and ventral mPFC in freely moving mice. Our results provide direct evidence that there is a significant reduction in the spike rates of vmPFC neurons in mice subjected to social defeat stress. In addition, we discovered that a unique transient oscillatory pattern of 20–40 Hz frequency appears specifically in the vmPFC. The vmPFC-specific 20–40 Hz oscillations were affected by social defeat stress and restored by ketamine treatment.

## Results

### Chronic social defeat stress reduces vmPFC neuronal spikes

**Figure 1:**
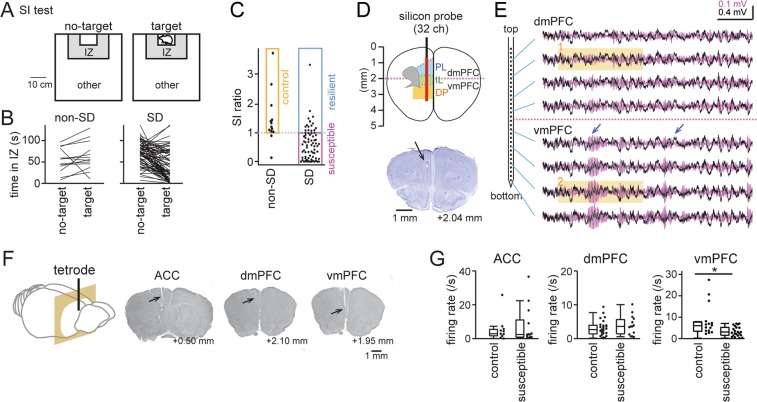
The vmPFC, but not the dmPFC, shows pronounced decreases in neuronal spike rates. (**A**) Schematic illustration of the social interaction (SI) test showing the interaction zone (IZ) and the other region. (**B**) Time periods in which individual mice stayed in the IZ. Each line shows each mouse (*n* = 14 non-SD mice and 73 SD mice). (**C**) For each animal, an SI ratio was computed as the ratio of occupancy time in the IZ region in the target session to that in the no-target session. Each dot represents an animal. Non-SD mice with a SI ratio of more than 1 are labeled as the orange region, termed as control mice, and SD mice with a SI ratio of less than and more than 1 were classified as stress-susceptible (magenta) and stress-resilient (cyan) mice, respectively. (**D**) (Top) A 32-site silicon probe was implanted along the dorsoventral axis of the mPFC. Recording sites (interval = 100 μm) are represented by a red line. (Bottom) Histological verification of the dorsal part of a recording site indicated by the arrow. **(E)** Representative simultaneous recordings of LFP signals obtained from the dmPFC and vmPFC using the silicon probe. Unfiltered and bandpass-filtered (20–40 Hz) LFP traces, including 20–40 Hz events (arrows), are superimposed and are shown in black and magenta, respectively. The shaded regions are magnified in Fig. 2A. The horizontal scalebar represents 500 ms. (**F**) Histological confirmation of tetrode recording locations in the ACC, dmPFC and vmPFC (indicated by arrows). **(G)** Comparison of neuronal firing rates in individual subcortical areas during SI tests in control and stress-susceptible mice (ACC, *n* = 11 and 13 cells; dmPFC, *n* = 32 and 15 cells; vmPFC, *n* = 15 and 24 cells). *P < 0.05, Mann-Whitney U test.

Mice were subjected to chronic social defeat (SD) stress for 10 consecutive days, termed as SD mice. Mice that were not subjected to SD stress were labeled as non-SD mice. We first assessed their stress susceptibility by comparing the time spent in the interaction zone (IZ) during no-target and target sessions in a social interaction (SI) test (Fig. 1A,B). First, we merged all data from all mice with and without electrodes implanted into the brain. No significant differences in the time periods at the IZ during the no-target session were found between non-SD and SD mice (*n* = 14 non-SD and 73 SD mice; *U* = 453.5, *Z* = 1.87, *P* = 0.061, Mann-Whitney U test), confirming that loading of SD alone had no prominent effects on the motivation of mice to explore around the IZ. On the other hand, individual mice showed pronounced differences in the time periods at the IZ during the target session. For each animal, an SI ratio was computed as the ratio of occupancy time in the interaction zone (IZ) in the target session to that in the no-target session (Fig. 1C). Out of the 14 non-SD mice, 12 mice had a SI ratio of more than 1 (orange region), which were further analyzed as control mice. Out of the 73 SD mice, 57 and 16 mice had a SI ratio of less than and more than 1, classified as stress-susceptible (magenta) and stress-resilient mice (cyan), respectively, confirming that our SD protocol could induce larger proportions of stress-susceptible mice. Out of these mice, electrophysiological recordings were obtained from 11 control mice and 17 stress-susceptible mice.

To examine subregional differences in neuronal activity patterns in the dmPFC, vmPFC, and anterior cingulate cortex (ACC), we implanted a silicon-based multielectrode array (32-site vertical linear spacing at 100 µm) (Fig. 1D,E; *n* = 4 naïve mice) or a tetrode array (Fig. 1F; *n* = 28 control and SD mice) throughout the dorsoventral axis of the mPFC. Here, the dmPFC was defined as the prelimbic (PL) region and the dorsal portion of the infralimbic (IL) region (up to 2 mm in depth), and the vmPFC was defined as the ventral portion of the IL region and the dorsopeduncular (DP) region. By collecting datasets from the extracellular multisite recordings, we analyzed subregional differences in the firing rates of individual neurons (Fig. 1G). Overall, the firing rates of individual vmPFC neurons in control and stress-susceptible mice differed significantly (*n* = 15 and 24 cells from 7 control and 10 stress-susceptible mice, respectively; *U* = 396, *Z* = 2.76, *P* = 0.0058, Mann-Whitney U test). This significant result was similar even when three cells with a firing rate of >10 Hz in control mice were removed (*n* = 12 and 24 cells from 7 control and 10 stress-susceptible mice, respectively; *U* = 282, *Z* = 2.00, *P* = 0.046). On the other hand, no significant differences were found in the firing rates of dmPFC and ACC neurons (dmPFC, *n* = 32 and 15 cells from 7 control and 10 stress-susceptible mice, respectively; *U* = 716, *Z* = 1.18, *P* = 0.24; ACC, *n* = 11 and 13 cells from 4 control and 7 stress-susceptible mice, respectively; *U* = 142, *Z* = 0.23, *P* = 0.82). These results provide direct evidence that a prominent reduction in neuronal excitability occurs specifically in vmPFC neurons after social defeat stress.

### Detection of 20–40 Hz events in vmPFC local field potential signals

**Figure 2:**
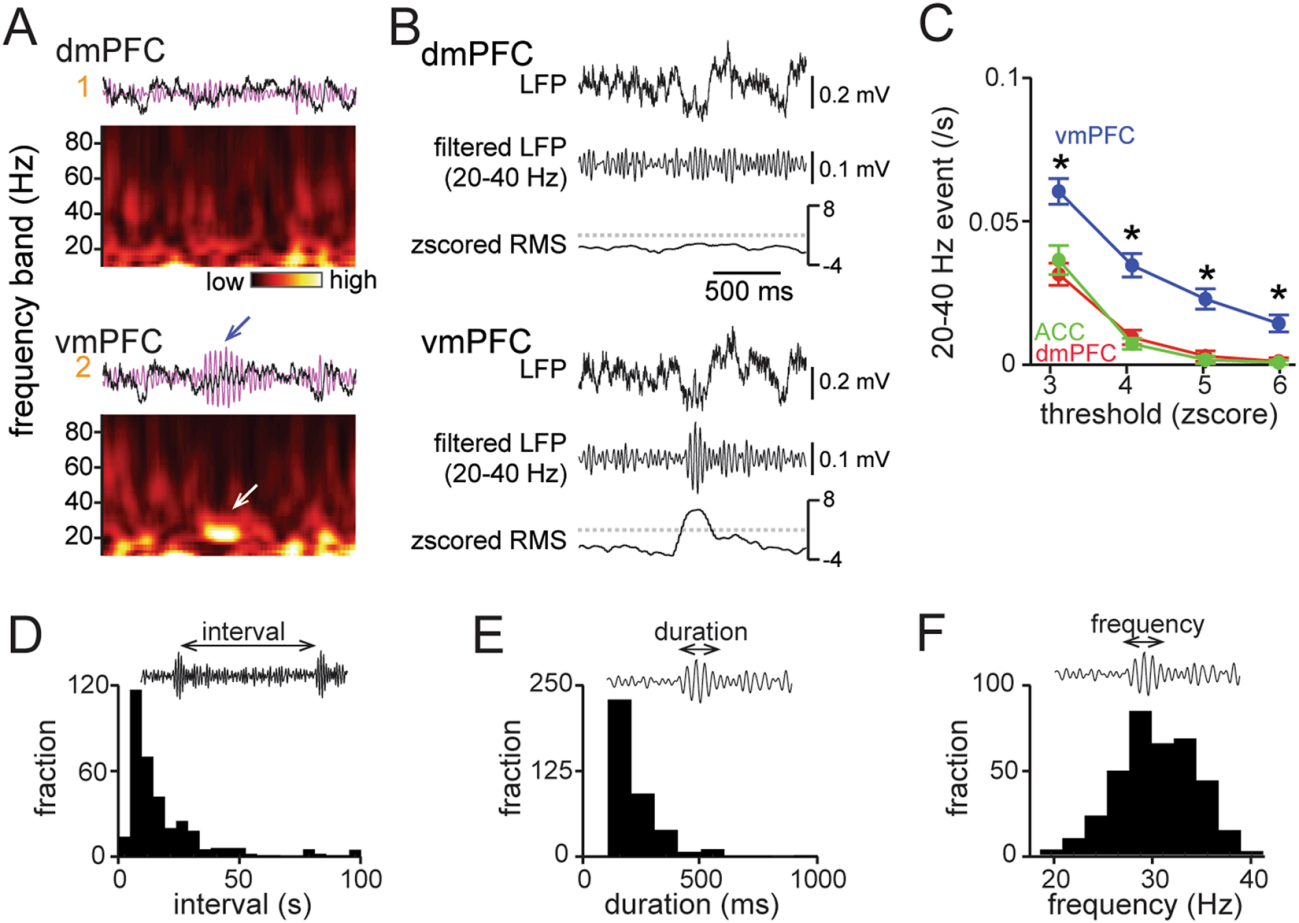
Physiological characteristics of vmPFC 20–40 Hz events in control mice. (**A**) LFP power spectrogram constructed from LFP traces magnified from the shaded regions in Fig. 1E. (B) Simultaneous LFP recordings from the dmPFC and vmPFC during the occurrence of a vmPFC 20–40 Hz event. Each panel shows typical unfiltered traces (top), 20–40 Hz-filtered LFP traces (middle), and the corresponding z-scored RMS amplitudes (bottom). (**C**) The average frequency of 20–40 Hz events during SI tests plotted against variable detection thresholds of z-scored amplitudes (*n* = 18 electrodes from 11 mice (vmPFC), 15 electrodes from 11 mice (dmPFC), and 11 electrodes from 8 mice (ACC)). *P < 0.05, Mann–Whitney U test followed by Bonferroni correction, vmPFC versus dmPFC and vmPFC versus ACC. (**D**) Distribution of vmPFC 20–40 Hz event intervals between two neighboring 20–40 Hz events (*n* = 352 intervals). (**E**,**F**) Distribution of duration (**E**) and oscillation frequency (**F**) of individual 20–40 Hz events (*n* = 382 events).

Through screening of local field potential (LFP) signals by band-pass filtering and wavelet analyses, we observed that short (typically < 0.5 s) bouts of 20–40 Hz oscillations specifically appeared in the vmPFC but not in the dmPFC (Figs 1E and 2A). All four mice tested using a silicon-based multielectrode array showed similar LFP patterns. To mathematically extract such transient increases in 20–40 Hz oscillations, termed 20–40 Hz events, we calculated the root-mean-square (RMS) of the band-pass (20–40 Hz) filtered LFP signals, which represents the absolute magnitude of the filtered LFP signals relative to the baseline. The RMS values were then z-scored (Fig. 2B). To determine the detection threshold for apparent vmPFC 20–40 Hz events, we tested varying thresholds from 3 to 6 (Fig. 2C; see Methods). At an amplitude threshold of 3 or higher, the incidence rate of 20–40 Hz events in the dmPFC and ACC decreased to nearly zero and was significantly lower than the incidence rate of these events in the vmPFC (vmPFC, *n* = 18 electrodes from 11 mice; dmPFC, *n* = 15 electrodes from 11 mice; ACC, *n* = 11 electrodes from 8 mice; *P < 0.05, Mann-Whitney U test followed by Bonferroni correction, vmPFC between dmPFC and vmPFC between ACC). These results verify that a detection threshold of 3 or higher is appropriate for extracting vmPFC-specific 20–40 Hz events, consistent with our intuitive impression. We here set an automatic detection threshold of vmPFC events of 4 in the following analyses. The distribution of inter-event intervals between neighboring vmPFC 20–40 Hz events (Fig. 2D; *n* = 382 events) shows that 48.9% and 71.9% of events occurred within 10 s and 20 s, respectively, after previous events. The distributions of the durations and the oscillatory frequencies of vmPFC 20–40 Hz events (*n* = 382 events) are shown in Fig. 2E,F, respectively.

### Transient decreases in vmPFC neuronal spike rates during vmPFC 20–40 Hz events

**Figure 3:**
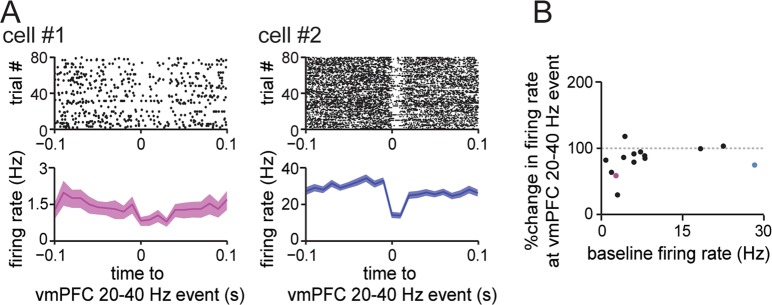
Modulation of vmPFC neuronal spikes by vmPFC 20–40 Hz events in control mice. **(A)** Two sample cells showing significant reductions in their firing rates during vmPFC 20–40 Hz events. (Top) A raster plot showing the spike patterns of a vmPFC neuron aligned to the centers of individual vmPFC 20–40 Hz events. Each dot indicates a spike. (Bottom) Average firing rate changes corresponding to the raster plots. (**B**) Relationship between baseline firing rate and percentage change in firing rate during vmPFC 20–40 Hz events (*n* = 14 cells). Each dot represents a vmPFC neuron. The magenta and blue dots in Fig. 3A show cell #1 and cell #2, respectively.

LFP oscillations, which represent the oscillatory activity patterns of neuronal networks, are locked to changes in neuronal spiking activity. We thus examined whether ongoing spike patterns of vmPFC neurons are altered during vmPFC 20–40 Hz events. Figure 3A shows raster plots of vmPFC 20–40 Hz event-triggered spike patterns obtained from two representative vmPFC neurons. Cell #1 shows significant reductions in its instantaneous firing rate during vmPFC 20–40 Hz events compared with baseline (out-of-event) spike rates (*t*_5_ = 2.99, *P* = 0.030, paired *t*-test). Cell #2 shows a more pronounced event-triggered decrease in spike frequency (*t*_5_ = 8.85, *P* = 3.1 × 10^−4^, paired *t*-test). Figure 3B summarizes the percentage of vmPFC 20–40 Hz event-triggered firing rates relative to the cells’ individual baseline firing rates at 1–5 s before the events. Of 14 vmPFC cells with a baseline firing rate of >0.5 Hz tested, four cells including the two cells shown in Fig. 3A showed significant changes; the majority of the other cells tended to decrease their firing rates during vmPFC 20–40 Hz events, but the decrease in rate was not statistically significant. These results demonstrate that a subset of vmPFC neurons, but not all vmPFC neurons, exhibit pronounced modulation of firing rates that appear as vmPFC 20–40 Hz events. These results suggest that 20–40 Hz events are an electrophysiological sign of the transient inhibition of certain cell populations in the vmPFC.

### SD mice show decreases in vmPFC 20–40 Hz events

**Figure 4:**
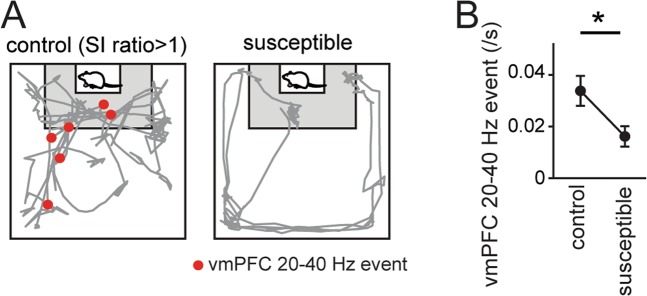
Reduction in vmPFC 20–40 Hz events in stress-susceptible mice. (**A**) Typical animal’s trajectories (gray) with vmPFC 20–40 Hz event locations superimposed (red dots). (**B**) Comparison of vmPFC 20–40 Hz events in control and stress-susceptible mice (*n* = 19 electrodes from 11 control mice and 28 electrodes from 17 stress-susceptible mice). *P < 0.05, Mann-Whitney U test.

We next tested whether vmPFC 20–40 Hz events are affected by social defeat stress. The representative data presented in Fig. 4A show that vmPFC 20–40 Hz events were considerably reduced in a stress-susceptible mouse compared with a control mouse. Overall, the incidence rates of vmPFC 20–40 Hz events observed during the SI tests in stress-susceptible mice were significantly lower than those in control mice (Fig. 4B; *n* = 19 electrodes from 11 control mice and 28 electrodes from 17 stress-susceptible mice; *U* = 518.5, *Z* = 3.33, *P = 8.6 × 10^−4^, Mann-Whitney U test). These results suggest that vmPFC 20–40 Hz events are sensitive to defeat stress, as are the changes in the firing rates of individual vmPFC neurons.

### Ketamine restores stress-induced reductions in vmPFC 20–40 Hz events

**Figure 5:**
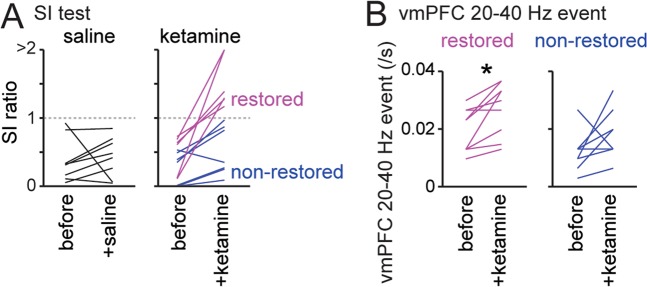
Mice showing a ketamine-induced increase in social interaction behavior exhibit a restoration of vmPFC 20–40 Hz events. (**A**) Changes in the SI ratios of stress-susceptible mice by saline (left, *n* = 8 mice) or ketamine (right, *n* = 13 mice) administration. Each line shows each mouse. Six mice (magenta) showed marked increases in SI ratios after ketamine administration (termed “restored”), whereas the other seven mice showed no changes in SI ratio (termed “non-restored”). (**B**) (Left) Increases in vmPFC 20–40 Hz event rates in the restored mice (*n* = 9 electrodes from 4 mice). (Right) No significant changes in vmPFC 20–40 Hz event rates were observed in the 5 non-restored mice that showed no changes in SI ratios after ketamine treatment (*n* = 10 electrodes from 5 mice). *P < 0.05, paired *t*-test.

After identifying a robust effect of SD stress on vmPFC 20–40 Hz events, we tested whether the decreased social interactions and vmPFC events are rescued by acute administration of ketamine. Ketamine has been reported to induce a rapid antidepressant response both in rodents^20,30–33^ and in treatment-resistant depressed patients^34,35^. Of 13 stress-susceptible mice tested, 6 mice markedly increased their SI ratios to more than 1 after systemic administration of ketamine (Fig. 5A; restored, magenta lines), whereas the other 7 mice did not exhibit such acute effects (Fig. 5A; non-restored, blue lines). All 8 stress-susceptible mice injected with saline did not exhibit SI ratios more than 1, confirming that saline injection alone did not induce pronounced effects. In the 4 restored animals, the incidence of vmPFC 20–40 Hz events increased significantly after ketamine treatment (Fig. 5B; *n* = 9 electrodes; t_8_ = 3.41, *P = 0.0092), whereas the incidence of vmPFC 20–40 Hz events in the 5 non-restored animals was unaltered (*n* = 10 electrodes; t_9_ = 2.24, *P* = 0.052). These results demonstrate that vmPFC 20–40 Hz events show changes that correlate with the degree of social interaction behavior.

## Discussion

In this study, we simultaneously recorded extracellular signals from the dorsal and ventral sides of the mPFC region and demonstrated that only the vmPFC, unlike the dmPFC and the ACC, exhibited significant decreases in the spike rates of individual neurons. Furthermore, we discovered that the vmPFC specifically generates unique, spatially localized 20–40 Hz events lasting for hundreds of milliseconds. Chronic social defeat stress-susceptible animals showed a reduction in the vmPFC 20–40 Hz events, and the decrease was restored specifically in mice that increased their social interactions after systemic administration of ketamine.

The dorsal portion of the prefrontal cortex, including the prelimbic (PL) cortex, and the ventral portion of the prefrontal cortex, including the infralimbic (IL) cortex, may have distinct functions in the regulation of mental states. While many early studies demonstrated anatomical subregional differences in the mPFC, the detailed electrophysiological characteristics of neurons in these subregions have not been fully elucidated. Our results provide direct evidence that the vmPFC is a specific subregion that is sensitive to stress and social interaction at single-spike levels. These results are consistent with previous morphological analyses in which it was shown that dendritic shrinkage and spine loss occur in pyramidal cells in the IL region^11–14^.

The vmPFC projects to a variety of subcortical areas^9^. A primate study demonstrated that the vmPFC, the ventral tegmental area (VTA), and the nucleus accumbens (NAc), are key components of the reward circuit^36^; this result was further supported by a rodent study showing that the IL region bidirectionally regulates the baseline excitability of individual neurons in the VTA^37^. Moreover, recent works have shown that the vmPFC-amygdala circuit plays a crucial role in the control of anxiety states and fear extinction^7,38^. Our results, together with these early findings, suggest that a stress-induced reduction in vmPFC neuronal activity might lead to dysfunctions of these subcortical regions that in turn lead to the dysregulation of fear responses and reward responsiveness.

Compared with well-characterized LFP oscillations with similar frequency bands such as gamma (25–100 Hz) and beta (20–30 Hz) oscillations^39–41^, the vmPFC 20–40 Hz events reported in this paper are relatively shorter in duration, lasting for hundreds of milliseconds, and lower in frequency (Fig. 2E,F). The mechanisms underlying vmPFC 20–40 Hz events remain an open question. Based on our results showing that decreases, rather than increases, in firing rates were dominant during the emergence of vmPFC 20–40 Hz events in a subset of vmPFC neurons, some inhibitory interneurons may be involved in such transient oscillatory events, as occurs in gamma oscillation^42,43^. Furthermore, some striking differences between the PL and IL regions may explain why the vmPFC but not the dmPFC generates apparent 20–40 Hz events: (1) the lamination in layer 2 and layer 3 is less clear in the IL region^44,45^; (2) the neuronal density of the IL region is lower than that of the PL region^44,45^; (3) the proportions of interneuron subtypes, including CCK-GABA and PV-GABA cells, differ in these two subregions^46^; and (4) the IL region receives stronger inputs from subcortical areas and the hippocampus^8^. In addition, previous studies using *in vitro* slice preparations have demonstrated that kainite-induced fast network oscillations with a frequency of 15–30 Hz in the IL and DP regions occurred with higher power than those observed in the PL region^47,48^, consistent with our results showing that the vmPFC possesses the ability to generate oscillations at this frequency range. Future investigations should extend this work to assess the functional roles of vmPFC 20–40 Hz events.

Administration of ketamine induces fast antidepressant actions in both rodents^20,30–33^ and human patients^34,35^. In particular, neuroimaging studies in humans demonstrate that ketamine increases glucose metabolic rates in the PFC^49^, and local injection of ketamine into the mouse IL region leads to acute antidepressant effects^20^, highlighting this brain area as a core region mediating antidepressant effects. Our results are consistent with these previous studies and provide direct neurophysiological evidence that the oscillatory activity of a stress-sensitive neuronal population, that is, the occurrence of vmPFC 20–40 Hz events, is associated with the effects of ketamine.

In conclusion, we provide physiological evidence that the vmPFC is a core subregion that shows changes in activity patterns at the single-neuron and neuronal population levels. The vmPFC-specific activity patterns described herein may serve as a biomarker of stress-sensitive social behavior and might be a therapeutic target in stress-induced mood disorders.

## Materials and Methods

### Approvals

This study was conducted in accordance with the NIH guidelines for the care and use of animals. The protocol was approved by the Experimental Animal Ethics Committee of the University of Tokyo (approval number: P29-14).

### Subjects

A total of 91 male C57BL/6J mice (8 weeks old) with preoperative weights of 20–30 g were used in this study. The animals were housed individually and maintained on a 12-h light/12-h dark schedule with lights on at 7:00 AM. All mice except the resident mice used in social defeat experiments (CLEA Japan, Tokyo, Japan) were purchased from SLC (Shizuoka, Japan). After at least 1 week of adaptation to the laboratory, the mice were used in experiments.

Eighty-seven mice were randomly assigned as 14 non-SD mice and 73 SD mice load with social defeat stress. Out of the 14 non-SD mice, 12 mice had a SI ratio of more than 1, termed control group. Out of the 73 SD mice, 57 and 16 mice were classified as stress-susceptible and stress-resilient mice in the SI test, respectively (Fig. 1C). Out of the 12 control and 57 stress-susceptible mice, 11 and 17 mice received surgery for implantation of a tetrode assembly and were used for recordings, respectively. Of the 57 stress-susceptible animals, 8 and 13 were administered saline and ketamine, respectively. In addition, 4 naïve animals were implanted with silicon probes.

### Chronic social defeat stress

SD mice were exposed to chronic social defeat stress as previously described^50–52^. At least 1 week before beginning the social defeat experiment, all resident CD1 mice more than 13 weeks of age were singly housed on one side of a home cage (termed the “resident area”; 42.5 cm × 26.6 cm × 15.5 cm). The cage was divided into two identical halves by a transparent Plexiglas partition (0.5 cm × 41.8 cm × 16.5 cm) with perforated holes, each with a diameter of 10 mm. The bedding in the resident area was left unchanged during the preoperative period. First, resident CD-1 mice were screened for social defeat experiments by introducing an intruder C57/BL6J mouse that was specifically used for screening into the home cage during three 3-min sessions on 3 subsequent days. Each session included a different intruder mouse. CD-1 mice were selected as aggressors in subsequent experiments based on three criteria: during the three 3-min sessions, (1) the mouse attacked in at least two consecutive sessions, (2) the latency to initial aggression was less than 60 s, and (3) the above two criteria were met for at least two consecutive days out of three test days. After screening, an experimental intruder mouse was exposed to social defeat stress by introducing it into the resident area for a 7–10-min interaction. The interaction period was immediately terminated if the intruder mouse had a wound and bleeding due to the attack, resulting in interaction periods of 7–10-min. After the physical contact, the intruder mouse was transferred across the partition and placed in the opposite compartment of the resident home cage for the following 24 h; this allowed the intruder mouse to have sensory contact with the resident mouse without physical contact. Over the following 10-day period, the intruder mouse was exposed to a new resident mouse so that the animals did not habituate the same residents.

Non-SD mice were pair housed in the same cage with one mouse per side of the same transparent partition with perforated holes, but it did not experience physical contact each other^50^.

### Surgery

A single surgery was performed in each animal to implant (i) a tetrode assembly or (ii) a silicon-based neural probe. In all experiments, the left side of the mPFC was targeted because its activity patterns have been reported to be associated with social behavior^53^ and antidepressant-like effects^24^. During all surgeries, the mice were anesthetized with isoflurane gas (1–3%). (i) An electrode assembly that consisted of 6–8 independently movable tetrodes was created using a 3-D printer (Form2, Formlabs) and stereotaxically implanted above the left dmPFC and vmPFC (1.94 mm anterior and 0.83 mm lateral to bregma) and the left anterior cingulate cortex (ACC) (1.0 mm anterior and 0.83 mm lateral to bregma) at an angle of 10°. The tetrodes were constructed from 17-μm-wide polyimide-coated platinum-iridium (90/10%) wires (California Fine Wire), and the electrode tips were plated with platinum to lower the electrode impedances to 200–250 kΩ. (ii) A silicon probe that consisted of 32 recording sites (A1 × 32–6 mm-100–177, NeuroNexus) was implanted at 1.94 mm anterior and 0.5 mm lateral to bregma. The probe was implanted so that the top recording site was located between the cortical surface and 500 μm below the cortical surface. In all surgeries, an additional incision was made at the incised neck area, and one EMG electrode (stainless-steel wires; AS 633, FEP Hookup Wire Stranded Stainless Steel, Cooner Wire Company) was sutured to the dorsal neck muscles. Ground electrodes were located on the cerebellum. The recording device was secured to the skull using stainless steel screws and dental cement (Re-fine Bright, Yamahachi Dental Mfg. Co.). The tetrodes were advanced to the targeted brain regions over a period of at least one week following surgery. After all surgical procedures were completed, anesthesia was discontinued, and the mice were allowed to awaken spontaneously. Following surgery, each animal was housed in a transparent Plexiglas cage with free access to water and food.

### Adjusting tetrode depth

The mouse was connected to the recording equipment via Cereplex M (Blackrock Microsystems), a digitally programmable amplifier, which was placed close to the animal’s head. The output of the headstage was conducted to the Cereplex Direct recording system (Blackrock  Microsystems), a data acquisition system, via a lightweight multiwire tether and a commutator. The depth of the electrodes was adjusted while the mouse rested in the home cage. Over a period of at least 10 days following surgery, the electrode tips were advanced up to 31.25–125 μm per day until 20–40 Hz oscillatory events were encountered in the IL, which was identified on the basis of LFP signals. The depth of the vmPFC was typically 2000–2500 μm. The tetrodes were then settled into the targeted area so that stable recordings were obtained.

### Electrophysiological recording

For recording electrophysiological signals, the EIB of the microdrive array was connected to a Cereplex M digital headstage (Blackrock Microsystems), and the digitized signals were transferred to a Cereplex Direct data acquisition system (Blackrock Microsystems). Electrical signals were sampled at 2 kHz and low-pass filtered at 500 Hz. The unit activity was amplified and bandpass filtered at 750 Hz to 6 kHz. Spike waveforms above a trigger threshold (50 μV) were time-stamped and recorded at 30 kHz in a time window of 1.6 ms. The animal’s moment-to-moment position was tracked at 15 Hz using a video camera attached to the ceiling. The frame rate of the movie was downsampled to 3 Hz, and the instantaneous speed of each frame was calculated based on the distance traveled within a frame (~333 ms).

### Social interaction test (SI test)

Social interaction tests were performed inside a dark room with a light intensity of 10 lux in a square-shaped box (39.3 cm × 39.3 cm) enclosed by walls 27 cm in height. An ABS-mesh cage (6.5 cm × 10 cm × 24 cm) was centered against one wall of the arena during all social interaction sessions. Each social interaction test included two 150-s sessions (separated by an intersession interval of 30 s) without and with the target CD-1 mouse present in the mesh cage; these sessions were termed “no-target” and “target” sessions, respectively. In the no-target session, a test C57BL/6 J mouse was placed in the box and allowed to freely explore the environment. The C57BL/6 J mouse was then removed from the box. In the 30-s break between sessions, the target CD-1 mouse was introduced into the mesh cage. The design of the cage allowed the animal to fit its snout and paws through the mesh cage but not to escape from the cage. In the target session, the same C57BL/6 J mouse was placed beside the wall opposite to the mesh cage. In each session, the time spent in the interaction zone (IZ), a 14.5 cm × 26 cm rectangular area extending 8 cm around the mesh cage was analyzed. The social interaction (SI) ratio was computed as the ratio of time spent in the interaction zone in the presence of the target to the time spent there in the absence of the target. Defeated mice with SI ratios less than 1 and more than 1 were classified as stress-susceptible and stress-resilient animals, respectively.

### Ketamine administration

In each mouse, after the first SI test, ketamine (20 mg/kg; Daiichi Sankyo Propharma) was intraperitoneally injected into the mice. One day after the ketamine administration, the second SI test was performed. As a control experiment, phosphate-buffered saline (pH 7.4) (PBS), termed as saline, was administrated similar to ketamine.

### Histological analysis and confirmation of electrode locations

The mice were overdosed with isoflurane, perfused intracardially with 4% paraformaldehyde in PBS and decapitated. After dissection, the brains were fixed overnight in 4% PFA and equilibrated with 20% and 30% sucrose in phosphate-buffered saline for an overnight each. Frozen coronal sections (40 μm) were cut using a microtome, and serial sections were mounted and processed for cresyl violet staining. For cresyl violet staining, the slices were rinsed in water, stained with cresyl violet, and coverslipped with Permount. The positions of all electrodes were confirmed by identifying the corresponding electrode tracks in histological tissue. Along the dorsoventral axis of the mPFC, recording sites at depths of less than 2 mm and more than 2 mm from the cortical surface were considered to represent the dmPFC and the vmPFC, respectively. In addition, the emergence of typical vmPFC 20–40 Hz events (see Fig. 2B) was utilized to estimate electrode locations in some cases.

### LFP analysis

To compute the time-frequency representation of LFP power, LFP signals were convolved using complex Morlet wavelet transformation at frequencies ranging from 1 to 250 Hz. The absolute power spectrum of the LFP during each 0.5-ms time window was calculated, and z-scores were computed for each frequency band across an entire analyzed period. For the detection of 20–40 Hz events, LFP traces were bandpass filtered at 20–40 Hz, and the root mean-square (RMS) power was calculated using a bin size of 100 ms. The threshold for 20–40 Hz event detection was set to 3–6 (Fig. 2C) and 4 (other figures) standard deviations above the mean with a duration of more than 200 ms.

### Spike unit analysis

Spike sorting was performed offline using the graphical cluster-cutting software Mclust^54^. Sleep recordings obtained before and after the behavioral paradigms were executed were included in the analysis to assure recording stability throughout the experiment and to identify cells that were silent during behavior. Clustering was performed manually in 2D projections of the multidimensional parameter space (i.e., comparisons between the waveform amplitudes, the peak-to-trough amplitude differences, the waveform energies, and the first principal component coefficient (PC1) of the energy-normalized waveform, each measured on the four channels of each tetrode). Only clusters that could be stably tracked across all behavioral sessions were considered to be the same cells and were included in our analysis. Our analysis may include not only excitatory pyramidal cells but also inhibitory interneurons that exhibited an average firing rate of more than 10 Hz^55^.

### Data analysis

All analyses were performed using Matlab (Mathworks). All data are presented as the mean ± standard error of the mean (SEM). All *P*-values reported were two-tailed. The null hypothesis was rejected at the *P* < 0.05 level unless otherwise specified.

## Data Availability

The datasets collected and analyzed are available from the corresponding author on request.
